# Cervical Spine Ischemic Stroke Complicated by Spastic Quadriparesis and Ogilvie Syndrome: A Case Report and Literature Review

**DOI:** 10.1155/2020/7197230

**Published:** 2020-07-15

**Authors:** Salman Assad, Justin Nolte, Dharampreet Singh, Samrina Hanif, Paul Ferguson

**Affiliations:** Department of Neurology, Marshall University School of Medicine, Huntington, WV, USA

## Abstract

Infarction or ischemia of the spinal cord is a rare entity and is often misdiagnosed as inflammatory myelopathy in acute settings. Atherosclerotic disease can affect spinal arteries, leading to cord ischemia with clinical presentation mixed with myelopathy. We present a case of a 66-year-old male who came to the hospital with unsteady gait and numbness of all extremities without associated pain for the past 48 hours. The neurological examination on admission directed the diagnosis towards myelopathy of the cervical spine. However, the initial magnetic resonance imaging (MRI) of the cervical spine demonstrated gliosis and restricted diffusion of the cord with multilevel neuroforaminal stenosis but without central canal stenosis or cord compression. The MRI brain, cerebrospinal fluid analysis, and rheumatologic evaluation were unremarkable. Four days into the clinical course, the patient developed weakness and spasticity of all extremities prompting further evaluation. Computed tomography angiography (CTA) scan of the head and neck revealed right vertebral artery occlusion and intracranial atherosclerotic disease. He was started on aspirin and clopidogrel for secondary risk reduction. The hospital course was further complicated by Ogilvie syndrome (OS), and the patient underwent uncomplicated cecostomy.

## 1. Introduction

Spinal cord ischemia is caused by a vascular interruption that can lead to cord dysfunction, ischemia, and infarction [1]. The blood supply of the spinal cord consists of anterior and posterior spinal arteries. The cervical cord lesions can typically present with either acute quadriparesis or paraparesis. Three vessels arising from vertebral arteries supply the spinal cord in the neck consisting of anterior and two posterior spinal arteries [2]. The manifestation of cord infarction is spontaneous with unknown etiology. Vascular aneurysm, dissection, and postsurgical complications should be ruled out when suspecting a cord ischemic stroke [3]. We present a unique and first-ever report of cryptogenic cervical cord acute ischemic infarction with late-onset development of Ogilvie syndrome.

## 2. Case Presentation

**Figure 1:**
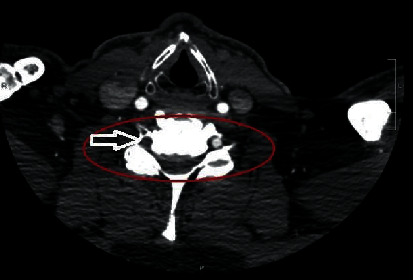
The computed tomography angiography (CTA) of the the head and neck showed right vertebral artery occlusion with moderate diffuse intracranial atherosclerosis.

**Figure 2:**
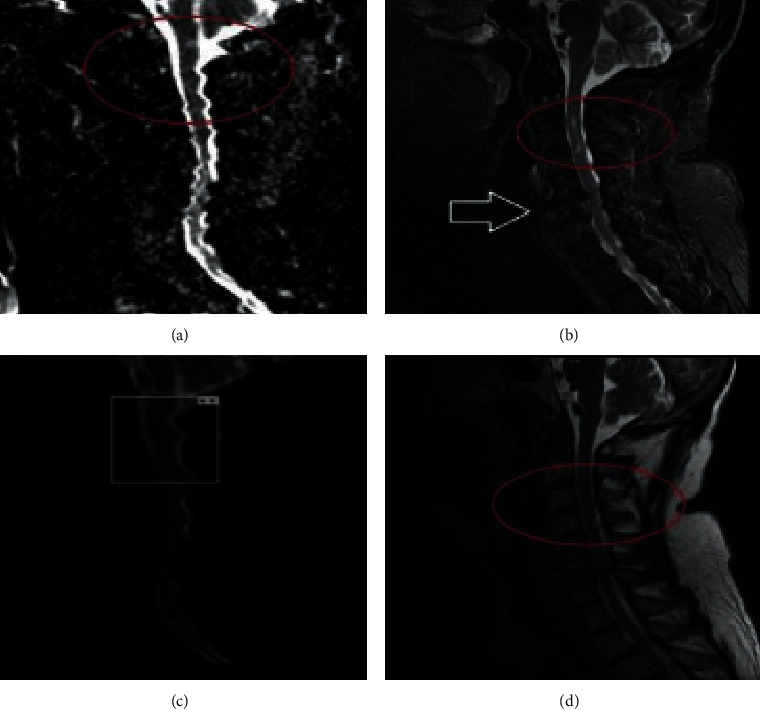
(a–d) MRI of the cervical spine with and without gadolinium demonstrated a restriction on diffusion-weighted imaging (DWI) (a) corresponding to the signal abnormality foci seen on short-TI inversion recovery (STIR) images (red circles in (b) and (d) and white marker image in (c)) and anterior compression from displaced cervical discs (white arrow in (b)).

A 66-year-old male with a past medical history of hypertension, hyperlipidemia, coronary artery disease, prostate cancer, and gastroesophageal reflux disease presented to the emergency department with intermittent symptoms of disequilibrium, gait instability, and progressive numbness of all extremities. The patient also developed hypotension and required vasopressors and intravenous fluid support after his neurologic symptomatology further evolved into weakness and spasticity. Laboratory investigation revealed hypercholesterolemia with normal thyroid-stimulating hormone, vitamin B12, and folate levels. Transthoracic echocardiography showed ejection fraction >55%, no wall abnormalities, and no patent foramen ovale. The MRI of the brain was unremarkable. The computed tomography angiography (CTA) of the head and neck showed right vertebral artery occlusion with moderate diffuse intracranial atherosclerosis (Figure 1). On day 2 of admission, the patient's weakness of all four extremities worsened and became acutely myelopathic with Babinski and Hoffman's sign in the setting of diffuse hyperreflexia and quadriparesis. An MRI of the cervical spine with and without gadolinium demonstrated a restriction on diffusion-weighted imaging (DWI) corresponding to the signal abnormality foci seen on short-TI inversion recovery (STIR) images (Figure 2).

He further developed urinary retention and Ogilvie's (acute colonic pseudo-obstruction) syndrome. The MRI of the thoracolumbar region did not reveal any abnormality. For spasticity, the patient was given baclofen 20 mg three times per day and tizanidine 2 mg every 8 hours. For Ogilvie's syndrome, cecostomy was performed by the surgery team. The lumbar puncture and cerebrospinal fluid analysis showed normal IgG index 0.6 (normal range 0.0–0.7), high myelin basic protein 12 (normal range 0–1.2 nanograms/milliliter (ng/ml)), no oligo clonal bands, negative neuromyelitis optica spectrum disorders (NMOSD) antibody, and negative myelin oligodendrocyte glycoprotein (MOG) antibody. The blood cultures, urine cultures, and CSF diagnostic workup for viral, bacterial, or fungal infections were noncontributory. The patient was started on subcutaneous heparin for deep venous thrombosis prophylaxis, aspirin 81 mg daily, clopidogrel 75 mg daily, and atorvastatin 40 mg daily for secondary risk reduction. The patient was discharged to a rehabilitation facility.

## 3. Discussion

The onset of spinal cord ischemia or infarction symptomatology is usually abrupt like that noted in cerebral ischemia. A diffuse atherosclerotic process can affect spinal arteries and can lead to focal or nonfocal neurological deficits [3]. Cigarette smoking, uncontrolled hypertension, diabetes, positive family history of vascular insults, and hyperlipidemia are the major risk factors for spinal cord infarction [4]. The vertebrobasilar insufficiency can present with a multitude of symptoms such as bilateral body weakness, headaches, vomiting, diplopia, blindness, dizziness, and gait instability [5]. The symptoms of bladder dysfunction, quadriparesis, bilateral loss of pain, temperature sensation with intact proprioception, and vibration sensation are indicative of anterior spinal artery (ASA) syndrome [5]. The patient in our case has occluded right nondominant vertebral artery indicative of vertebrobasilar insufficiency. The functional outcome with spinal cord infarction is promising due to the noninvolvement of cognitive deficits [6]. Elzamly et al. reported a case of posterior cervical cord infarction after the neuroendovascular intervention for vertebrobasilar insufficiency [7]. It is imperative to mention that we did not consider thrombolysis in our case as cord infarction might have happened due to hypotensive episode. The patient in our report also developed neurogenic bladder and Ogilvie syndrome (colonic pseudo-obstruction), which is usually common with lesions below thoracic spine vertebra level 10 (T10) as reported by Korsten et al. [8].

Davda and Osman also mentioned a similar anterior spinal cord infarct with an affected spinothalamic tract involving the pain and temperature sensation [9]. This finding is in contrast to our report where both vibration and fine touch with pain and temperature sensations were affected, highlighting the involvement of both anterior and posterior spinal arteries. Ota et al. mentioned a patient who initially presented with sudden onset quadriplegia with an MRI cervical spine indicative of myelopathy [10]. After almost one week of the worsening of neurological symptoms, DWI and ADC images of the MRI cervical spine were obtained that showed acute cord infarction [10]. On the other hand, our patient developed spastic quadriplegia almost after 10 days of onset with intermittent episodes of unsteady gait. Müller et al. and Restrepo et al. mentioned the prompt recovery within few days of ASA syndrome in patients presented within 3 hours of symptoms and treated with intravenous recombinant tissue plasminogen activator (tPA) [4, 11]. It is also necessary to look for vascular dissection, aneurysm, or malformations before giving tPA [11]. The fluid resuscitation and use of dual antiplatelet therapy (DAPT) with statins were found to be associated with good outcomes in ASA syndrome patients [4]. The role of a mechanical thrombectomy or neuroendovascular procedures in such cases is still debatable and will require future studies.

The first limitation of our case presentation was not taking into consideration the magnetic resonance angiography (MRA) or conventional angiogram of the cervical spine in the beginning. The second limitation was not ordering a repeat MRI or MRA of the thoracolumbar spine on day 14 of hospitalization after the patient developed Ogilvie syndrome to rule out thoracolumbar infarction. However, the CTA of the head and neck was done and vascular dissection or aneurysm was not reported which in the past has been associated with spinal cord vascular syndromes. We conclude that the timely diagnosis of cord ischemia is important and MRI with DWI/ADC, MRA, or conventional angiogram should be considered in such cases. If within the window, tPA can be considered followed by DAPT for 3–6 months based on clinical discretion until clinical trials are available in the literature.
